# Negative pressure wound therapy on a musculocutaneous free flap in no skin edge wound bed: A case report

**DOI:** 10.1016/j.ijscr.2025.110878

**Published:** 2025-01-13

**Authors:** Camilla Mensel, Jacob Juel, Birgitte Jul Kiil, Hans Henrik Rohden Nielsen

**Affiliations:** a Department of Plastic and Breast Surgery, Aarhus University Hospital, Denmark; b Department of Plastic and Breast Surgery, Aalborg University Hospital, Denmark

**Keywords:** Microsurgery, Negative pressure wound therapy, Necrotising soft tissue infection

## Abstract

**Introduction:**

Necrotising soft tissue infection (NSTI) is an exceptionally dangerous infectious disease targeting soft tissues with high mortality as well as morbidity.

The aim of reconstructive surgery after initial debridement is to maintain function as well as to achieve a satisfactory cosmetic result.

**Presentation of case:**

A 50-year-old male presented with necrotising soft tissue infection on the thorax and left upper arm following mastectomy for breast cancer. He underwent aggressive debridement and was left with a large complicated soft tissue defect on the thorax, abdomen and left axilla with a wound bed consisting of exposed bone, nerves and vessels. There were close to no adjacent skin edges to fixate the flap, due to the size of the defect. Reconstruction with a free musculocutaneous latissimus dorsi (LD) and split skin grafting was performed. Vacuum therapy was applied immediately over the free flap and the vascular pedicle, as well as the skin graft.

**Discussion:**

Application of NPWT to the entire reconstructed area, including the free flap, in terms of achieving better and faster healing, is somewhat novel. Applying negative pressure wound therapy (NPWT) on free flaps is not a new practice but it has mostly been used as incisional therapy or when complications have occurred.

**Conclusion:**

Our case shows a successful microsurgical reconstruction in a challenging area, with the direct application of NPWT perioperatively, without compromising flap survival, and with good patient outcome.

## Highlights

• Demonstrates the safe and effective application of NPWT directly on a free flap.
• Uses free LD flap to cover a large defect in a no skin edge wound bed.
• Achieves excellent flap survival and restoration of shoulder mobility.

## 1 Introduction

Necrotising soft tissue infection (NSTI) is an exceptionally dangerous infectious disease targeting soft tissues with high mortality and morbidity. It is often caused by streptococcus bacteria and can afflict a broad range of patients, disregarding age or comorbidities. Infections travel along soft tissue. Treatment is aggressive surgical debridement followed by multidisciplinary therapy [1,2]. The rising incidence of NSTI as well as improved diagnostics and treatment, have resulted in better survival rates, therefore increasing need for reconstruction after debridement [1,2].

The aim of reconstructive surgery after initial debridement is to maintain function and achieve a satisfactory cosmetic result. Split skin grafting (SSG) is the workhorse procedure in re-establishing the necessary coverage after skin excision, but is not always sufficient in terms of preserving mobility and the necessary covering of blood vessels, nerves, bones, tendons and joints. This calls for transfer of elastic soft tissue. Local flap reconstructions are usually not viable in cases concerning NSTI due to the wide resection of surrounding soft tissue. Free flap transfer may be necessary to accomplish an optimal reconstructive outcome [3].

Reconstruction with free flaps is a well-integrated part of reconstruction in surgical centres today. Complications, although rare, can be severe and include partial flap necrosis or total flap loss. Negative pressure wound therapy (NPWT) postoperatively has been investigated as a way of preventing these complications along with early preoperative optimization of the patient [4,5].

In theory, NPWT can put strain on the vascular pedicle and compromise the microcirculation within the flap by compression as well as obstructing the monitoring of the free flap [6,7].

The literature regarding the subject is sparse. We explored the use of free flap transfer in coverage of a large, complicated defect after NSTI, as well as the application of NPWT on a free flap in the postoperative phase for fixation.

The work has been reported in line with the SCARE criteria [8].

## 2 Case

**Fig. 1:**
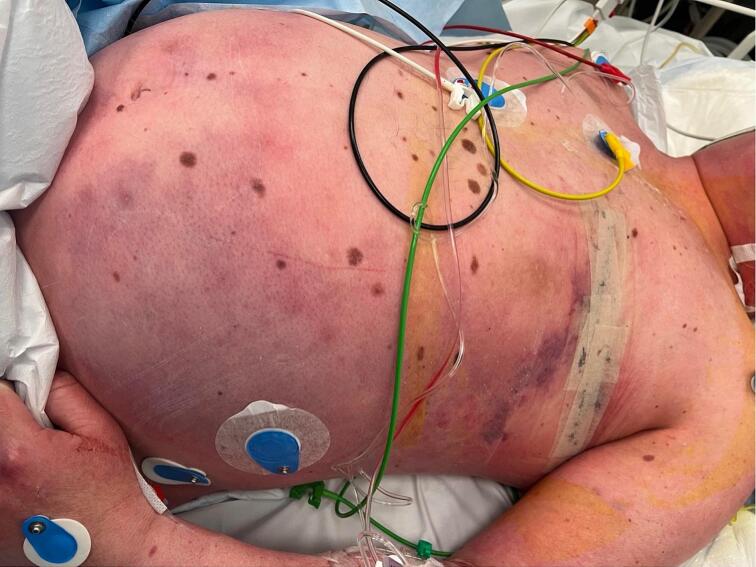
NSTI before debridement.

**Fig. 2:**
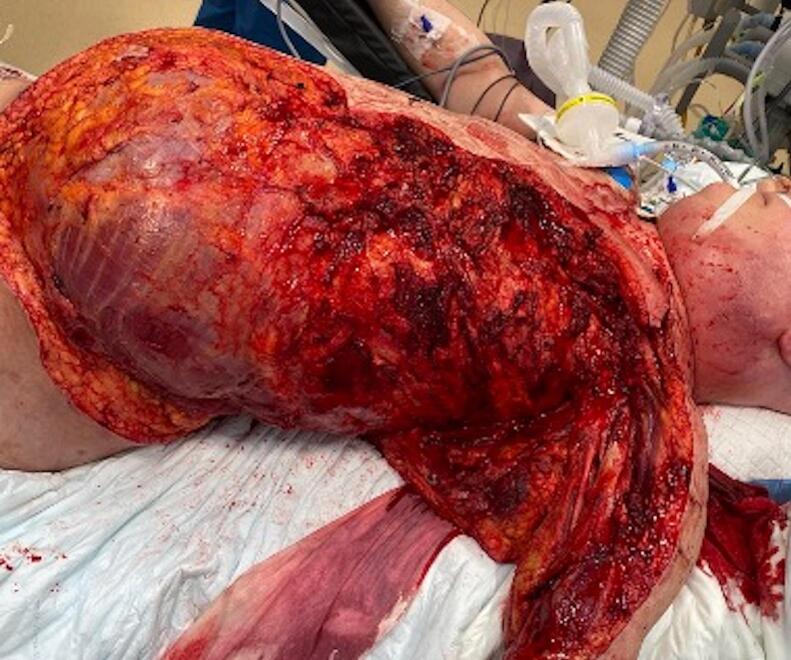
After debridement.

A 50-year-old male underwent left-sided mastectomy with axillary dissection due to breast cancer. He had a large ulcerating tumour of seven centimetres removed, as well as three non-suspicious axillary lymph nodes. No perioperative antibiotics were administered. One day postoperatively the patient was admitted to the emergency department with symptoms of respiratory distress, hypotension and cyanosis. He was transferred to the Department of Orthopaedics and diagnosed with NSTI (Fig. 1). Piperacillin/tazobactam was administered initially and substituted with meropenem and clindamycin. Acute surgical debridement was performed with resection of soft tissue of the left hemithorax, abdomen, neck, and upper arm including the pectoral muscles, the left side of the latissimus dorsi, resulting in exposed costa and periosteum, vessels and nerves in the axilla in an area of roughly 20 × 20 centimetres (Fig. 2). Primarily, the subsequent defect was treated with continuous NPWT and intravenous antibiotics (piperacillin/tazobactam) for 14 days. Initially, NPWT was changed every four to five days. Surgically, the main concern was the exposed nerves and vessels in the axilla, as well as the need for preservation of mobility in the left shoulder. The plastic surgical team was involved during the first week after primary debridement. NPWT treatment was prolonged to three weeks, to stabilize the patient and optimize the healing potential of the defect.

Three weeks after initial debridement, the patient had developed adequate granulation tissue for split-thickness skin grafting (SSG), and a small test graft was applied to the defect, in order to confirm the readiness of the wound bed and the healing potential. Final surgery was performed five weeks after admission.

**Fig. 3:**
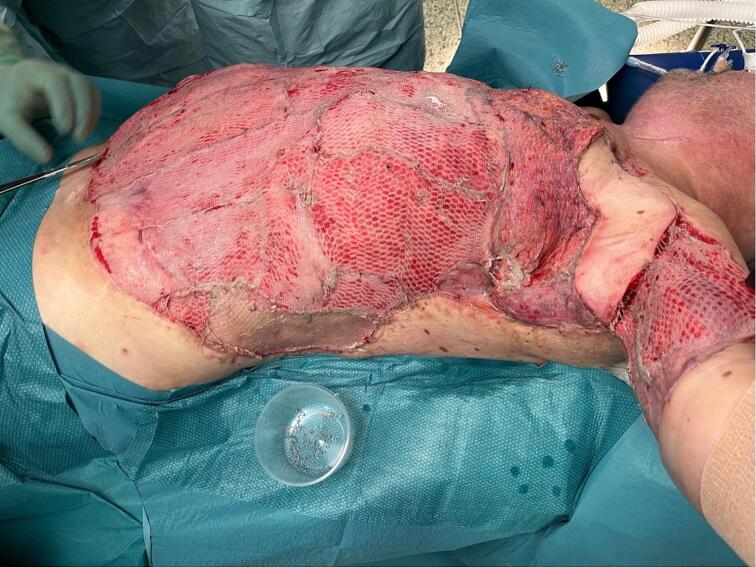
After reconstruction.

**Fig. 4:**
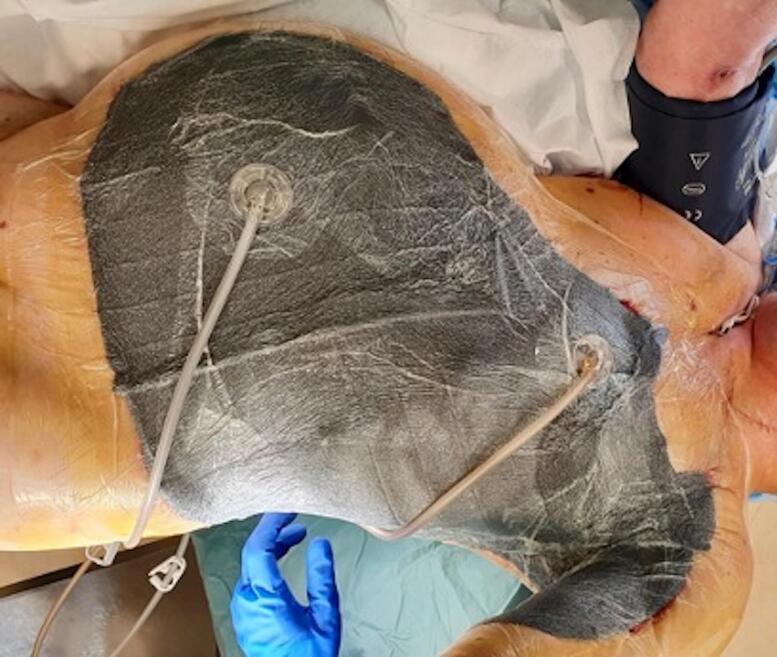
After application of NPWT.

The contralateral free partial latissimus dorsi (LD) flap was transferred to the left axilla for protection of the vessels and nerves and SSG was applied to the remaining defect (Fig. 3). The flap was raised with a large skin island of 25 × 5 cm to preserve mobility in the shoulder. The pedicle was anastomosed to the left internal mammary vessels in intercostal space 3. The recipient vessels were intentionally chosen to be at a distance from the defect, to reach vessels not affected by the infection, minimizing the risk of thrombosis. The vessels were anastomosed with 9–0 nylon sutures and the flap had an ischaemia time of 45 min. The most humeral part of the LD-muscle was split and used as coverage over the exposed vessels in the axilla and the vascular pedicle. The operation went as planned with no complications. To facilitate fixation of the skin graft and free flap, continuous NPWT was applied directly on the free flap and its pedicle - as well as on the SSGs - with a pressure of 80 mmHg (Fig. 4). A reduced pressure of 80 mmHg was chosen instead of 125 mmHg to minimize risk of compression of the vascular pedicle. The free flap was monitored every hour in the first five postoperative days, by trained microvascular nurses, through a small hole in the foam dressing, of 2 cm. The left arm was kept abducted 30° to avoid pressure on the flap.

**Fig. 5:**
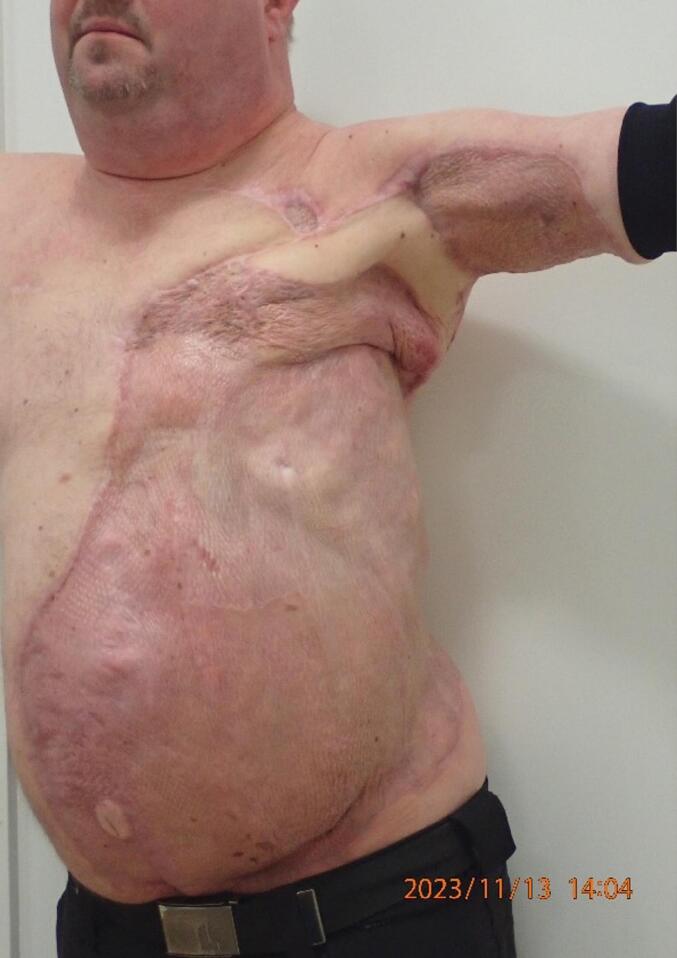
Nine months postoperative.

On the first change of the bandages on the fifth postoperative day, the flap was fully vital without oedema and without signs of partial necrosis or venous congestion. A take rate of more than 95 % of the SSG was confirmed, even on the exposed costa and periosteum. Subsequent treatment was open wound care with changes twice a day, including saltwater baths and exposure 30 min at a time. After 15 days of admission with wound care and physiotherapy, the patient was discharged to his own home, with mobility in the left shoulder joint of 70° abduction and 90° flexion. This increased to a range of motion of 100° in shoulder abduction nine months after resection (Fig. 5). The patient reported no experience of pain regarding the axilla or the areas with SSG and was able to return to work. The self-reported quality of life score was 90 out of 100, and most prominent complaint were fatigue.

## 3 Discussion

This case report presents a solution for covering a large, complex defect, requiring flap coverage, with minimal adjacent skin edges for fixating the flap. The safety and potential risks of applying NPWT directly on a free flap and its vascular pedicle was evaluated, focusing on its implications for flap vitality and wound healing.

Using an SSG across the entire defect was considered but deemed unsuitable due to poor graft take on exposed tendon and bone and the risk of vessel rupture, with large morbidity or possibly fatal outcome.

Local flaps were not viable due to the defect size leaving a free flap transfer as the sole option, preferably including both skin and muscle, in order to not only increase chances of flap attachment to the raw wound bed, but also to maintain elasticity [9]. The large defect consisted of a wound bed with a lack of clearly defined wound edges. An LD-flap was chosen for its viability, size and elasticity, with immediate NPWT applied to fixate the flap to the wound bed. The muscle protected the exposed axillary vessels from NPWT pressure, ensuring flap vitality and minimal oedema upon NPWT termination.

With challenging reconstructions, a close cooperation between the Department of Orthopaedics and Plastic Surgery before surgery is vital, optimizing the underlying infection, the following infection-mediated thrombogenic phase, and nutritional status. Therefore, microsurgical reconstruction should most optimally take place in the subacute phase after two to four weeks [4,5].

We believe that the perioperative direct application of NPWT contributed to the viability of the reconstruction. NPWT has several advantages, and the application directly on a free flap is not a contraindication, and both the arterial and venous anastomosis can withhold the pressure, ensuring viability of an otherwise large microvascular reconstruction.

Application of NPWT to the entire reconstructed area, including the free flap, in terms of achieving better and faster healing, is somewhat novel. Applying NPWT on free flaps is not a new practice but it has mostly been used as incisional therapy or when complications have occurred [7,10,11]. New studies further support the safety of application of NPWT directly on free flaps in the means of enhancing and optimizing healing [12]. Despite successful application in a clinical setting in many cases, it is important to remember the reason as to why NPWT needs to be applied to free flaps with caution [13]. The challenge is to avoid unnecessary stress on the anastomoses, which is why the NPWT pressure was set to 80 mmHg. The amount of granulation tissue is not critically superior in higher pressures levels, and therefore this relatively low vacuous pressure was deemed acceptable [14].

It had that further advantage that it made wound care on the ward easier for the nurses, since there were no raw wound surfaces and the patient could easier be mobilized. All this resulted in the ward being able to discharge the patient only 15 days after the reconstruction.

## 4 Conclusion

Our case demonstrates successful microsurgical reconstruction in a challenging area, using perioperative NPWT without compromising flap survival, and achieving good patient outcome. While further studies are needed to confirm NPWT's efficacy in free flap reconstructions, our case suggests it may support flap fixation and vitality without compromising the vascular pedicle.

## Consent

Written informed consent was obtained from the patient for publication of this case report and accompanying images. A copy of the written consent is available for review by the Editor-in-Chief of this journal on request.

## Ethical approval

Case reports, as this, are exempt from ethical approval at our institution. It is only required to have written consent from the patient, which have been obtained.

## Sources of funding

None.

## Declaration of competing interest

None.
